# Clinical significance of lipid pathway-targeted therapy in breast cancer

**DOI:** 10.3389/fphar.2024.1514811

**Published:** 2025-01-06

**Authors:** Dan Li, Pengcheng Jin, Yiqi Cai, Shijie Wu, Xianan Guo, Zhiyun Zhang, Kexin Liu, Panni Li, Yue Hu, Yunxiang Zhou

**Affiliations:** ^1^ Department of Breast Surgery and Oncology, The Second Affiliated Hospital, Zhejiang University School of Medicine, Hangzhou, Zhejiang, China; ^2^ Cancer Institute (Key Laboratory of Cancer Prevention and Intervention, China National Ministry of Education), The Second Affiliated Hospital, Zhejiang University School of Medicine, Hangzhou, China; ^3^ Department of Surgical Oncology, Linhai Branch, The Second Affiliated Hospital, Zhejiang University School of Medicine, Taizhou, Zhejiang, China

**Keywords:** breast cancer, lipid metabolism reprogramming, clinical trial, targeted therapy, predictive biomarkers, hypothesis

## Abstract

Globally, breast cancer represents the most common cancer and the primary cause of death by cancer in women. Lipids are crucial in human physiology, serving as vital energy reserves, structural elements of biological membranes, and essential signaling molecules. The metabolic reprogramming of lipid pathways has emerged as a critical factor in breast cancer progression, drug resistance, and patient prognosis. In this study, we delve into the clinical implications of lipid pathway-targeted therapy in breast cancer. We highlight key enzymes and potential therapeutic targets involved in lipid metabolism reprogramming, and their associations with cancer progression and treatment outcomes. Furthermore, we detail the clinical trials exploring the anticancer and cancer chemopreventive activity of therapies targeting these molecules. However, the clinical efficacy of these therapies remains controversial, highlighting the urgent need for predictive biomarkers to identify patient subpopulations likely to benefit from such treatment. We propose the Selective Lipid Metabolism Therapy Benefit Hypothesis, emphasizing the importance of personalized medicine in optimizing lipid pathway-targeted therapy for breast cancer patients.

## 1 Introduction

Globally, breast cancer represents the most common cancer and the primary cause of death by cancer in women (Bray et al., 2024). China has 357,200 new cases of breast cancer each year, posing a serious threat to the lives and health of a large number of women (Zheng et al., 2024). In spite of the progress made in diagnostic techniques and therapeutic interventions, a considerable proportion of patients encounter relapse subsequent to their initial course of treatment, resulting in diminished overall survival (OS) rates and a compromised quality of life (DeSantis et al., 2019; Zhou et al., 2023; Garcia-Martinez et al., 2021). This notable public health challenge underscores the urgency to explore further into the intricacies of breast cancer development and actively seek out promising therapeutic avenues.

Lipids empower diverse cellular life activities, constituting one of the three key energy sources. Furthermore, lipids are crucial in the constitution of cell membranes and function as pivotal signaling molecules (Xiao et al., 2024). Recent studies have revealed alterations in lipid metabolism within tumor cells and microenvironment, which furnish a robust substrate for tumor cell proliferation during therapy and drive the development of therapy resistance (Ligorio et al., 2021; Cheng et al., 2018; Bian et al., 2021; Liu et al., 2024). Correspondingly, the significance of lipid metabolism reprogramming in breast cancer is gradually coming to light (Zipinotti Dos Santos et al., 2023; Nelson et al., 2014; Guo et al., 2020; Bacci et al., 2021; Wang et al., 2024). Increasing evidence suggests that lipid remodeling plays a vital role in conferring drug resistance to breast cancer cells via mechanisms involving ferroptosis, immune escape, endoplasmic reticulum stress, and stemness (Zhang et al., 2019; Havas et al., 2017; Wang et al., 2018; Luis et al., 2021). Consequently, targeting lipid metabolism emerges as a promising therapeutic avenue to tackle resistance to conventional treatments. Notably, lipids occupy pivotal roles in maintaining normal physiological functions (Petrenko et al., 2023). Therefore, prior to contemplating systemic therapies aimed at lipid metabolism as therapeutic modalities for breast cancer, it is imperative to achieve a thorough comprehension of lipid functions within both cancer cells and normal cells, as well as their interplay.

In this study, we present an overview of the physiological processes underlying lipid metabolism and its remodeling in breast cancer. We have also summarized potential therapeutic targets within the lipid metabolism remodeling. Furthermore, we offer a detailed assessment of the evidence derived from both completed and ongoing clinical trials, encompassing those targeting lipid metabolism directly for anticancer effects, those utilizing lipid metabolism modulation as adjunctive therapy to mitigate the toxicity of traditional treatments, and those employing it as a prophylactic measure to prevent breast cancer occurrence. Despite the variability observed in the findings, specific breast cancer patients may indeed benefit from therapies that regulate the lipid pathway (Serageldin et al., 2024; El-Khayat et al., 2021; Kamal et al., 2024; Yulian et al., 2021; Dewidar et al., 2022; Yulian et al., 2023; Serageldin et al., 2023). Consequently, we propose the Selective Lipid Metabolism Therapy Benefit Hypothesis, suggesting that lipid pathway-targeted therapy may confer benefits to a selected group of patients. This hypothesis can aid in better understanding the role of lipid metabolism reprogramming in breast cancer, facilitating the discovery of more stable biomarkers that can predict treatment response, and thereby enabling effective clinical translation.

## 2 Physiological processes and key molecules of lipid metabolism

**FIGURE 1:**
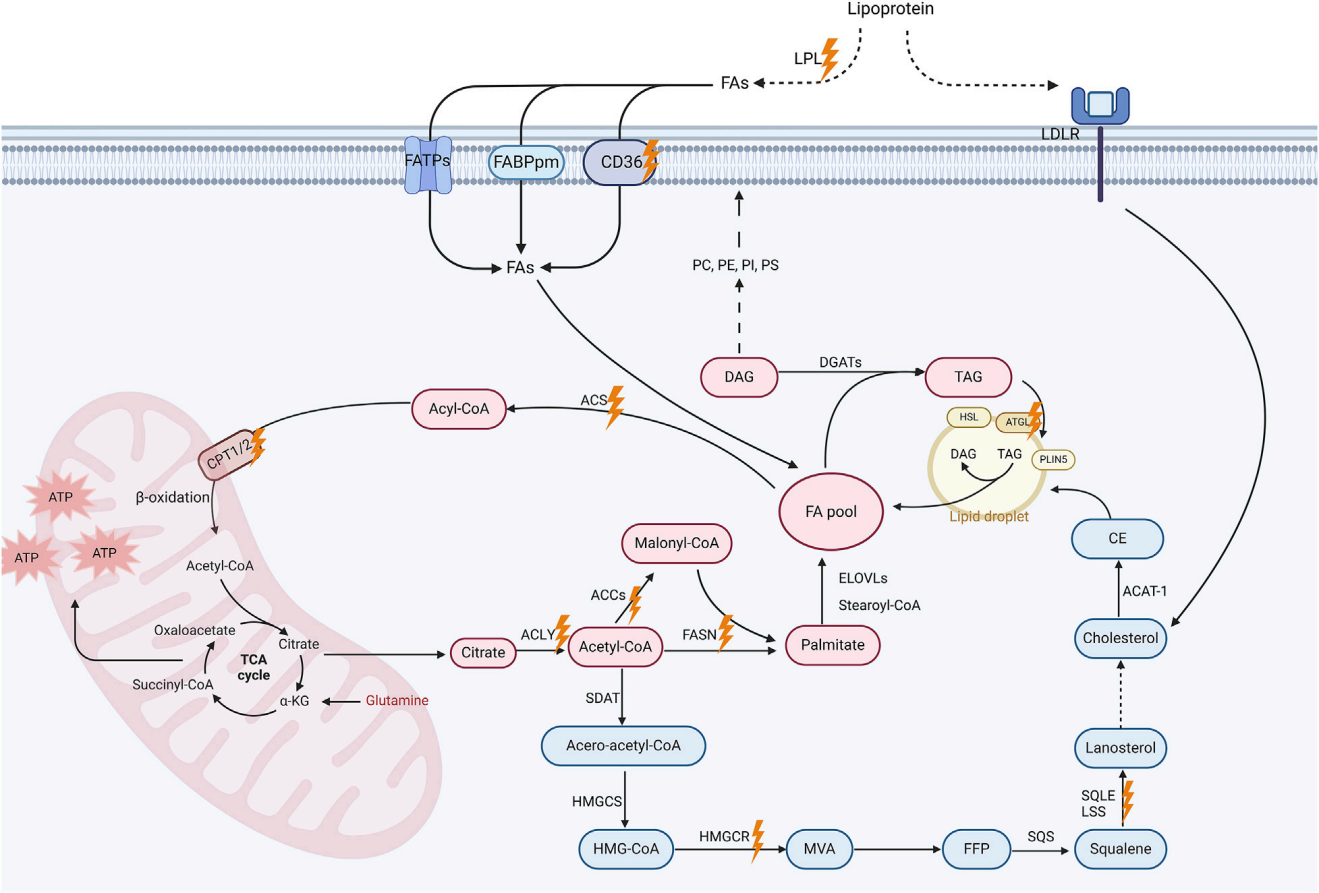
Lipid metabolism pathways and promising targets for breast cancer treatment. This schematic diagram illustrates the key processes and molecular involved in lipid metabolism, including fatty acid (FA) uptake, *De novo* lipogenesis (DNL), FA oxidation (FAO) and cholesterol biosynthesis. Extracellular triglycerides are hydrolyzed by lipoprotein lipase (LPL), releasing free FAs for cellular uptake via membrane-associated proteins such as CD36, fatty acid transport protein (FATP), and plasma membrane fatty acid-binding protein (FABP). Intracellularly, acetyl-CoA generated from FAs or glucose enters the tricarboxylic acid (TCA) cycle to produce energy in mitochondria. Acetyl-CoA also serve as a substrate for DNL. This metabolic pathway leads to the synthesis of malonyl-CoA and, ultimately, palmitate, which can subsequently undergo elongation and desaturation processes to yield a diverse array of FAs. These FAs can be modified to form diverse lipids including diacylglycerols (DAGs) and triacylglycerols (TAGs). TAGs are stored in lipid droplets, which regulate lipid storage and release under metabolic demands. Additionally, the cholesterol biosynthesis pathway, which is initiated by acetyl-CoA, proceeds through the formation of intermediate compounds such as farnesyl pyrophosphate (FPP) and squalene, eventually leading to the production of cholesterol. Cholesterol contributes to membrane fluidity and steroid hormone synthesis, with excess cholesterol stored as cholesterol esters in lipid droplets. Importantly, these metabolic pathways altered in breast cancer cells, which is likely associated with cancer progression. Promising therapeutic targets are marked with orange lightning symbols. Created in https://BioRender.com.

Lipids constitute one of the three key energy sources for cells, vital components of cell membranes, and essential signaling molecules, holding a paramount position as fundamental constituents within the physiological machinery of the body (Xiao et al., 2024). Over a thousand distinct lipid types have been identified within living cells, such as fatty acids (FAs), cholesterol, phospholipids, and others (Corradi et al., 2019). Understanding the physiological processes and key molecules of lipid metabolism in normal cells is crucial to grasping how it impacts the behavior of malignant cells and their therapeutic potential as targets (Figure 1).

The essential details of the biochemical processes underlying lipid metabolism have been comprehensively discussed elsewhere (Yu et al., 2021). Briefly, FA uptake is facilitated by lipoprotein lipase (LPL)-mediated extracellular hydrolysis of triglycerides into free FAs and glycerol, with CD36, among other membrane-associated proteins, playing a pivotal role in cellular FA transport (Ligorio et al., 2021; Abumrad et al., 2021; Samovski et al., 2023). *De novo* lipogenesis (DNL, i.e., *de novo* FA synthesis) occurs in the cytoplasm, utilizing acetyl-coenzyme A (acetyl-CoA) as the base stock. Key enzymes in this process include adenosine triphosphate (ATP)-citrate lyase (ACLY), acetyl-CoA carboxylase (ACC), and FA synthase (FASN), which catalyze the formation of cytosolic acetyl-CoA, malonyl-CoA, and long-chain saturated FAs (primarily palmitate), respectively (Vasseur and Guillaumond, 2022; Currie et al., 2013; Martin-Perez et al., 2022). FAs are able to undergo conversion into diverse types of lipids (Zipinotti Dos Santos et al., 2023; Dyall et al., 2022). For instance, FAs can be further metabolized to produce various glycerophospholipids, e.g., phosphatidylserine (PS), which are essential for membrane structure and function (Vance, 2015; Baenke et al., 2013; Baxter et al., 2015; Kopecka et al., 2020). FA oxidation (FAO) is a critical energy-producing process that occurs in four stages: FA activation, mitochondrial transfer, β-oxidation yielding acetyl-CoA, and acetyl-CoA entering the tricarboxylic acid (TCA) cycle. Enzymes such as acyl-CoA synthetase (ACS) and carnitine palmitoyltransferase (CPT) 1 take an active part in this process (Kemp et al., 2024; Li et al., 2017; Knottnerus et al., 2018; Carracedo et al., 2013).

Cholesterol stands out as another vital lipid, crucial for cellular function, impacting membrane fluidity, signal transduction, and steroid hormone synthesis (Luo et al., 2020). Cholesterol can be produced via the endogenous mevalonate (MVA) pathway or obtained extracellularly through transmembrane receptor proteins, primarily low density lipoprotein receptor (LDLR). Cholesterol biosynthesis involves a series of essential enzymes, including 3-hydroxy-3-methylglutaryl-coenzyme A (HMG-CoA) reductase (HMGCR), which serves as the rate-limiting enzyme in the MVA pathway, as well as squalene synthase (SQS), squalene epoxidase (SQLE), and lanosterol synthase (LSS) (Vasseur and Guillaumond, 2022; Luo et al., 2020). Excessive cholesterol is metabolized either into its primary metabolite, 27-hydroxycholesterol (27HC), or into cholesterol ester, which is then stored in lipid droplets (Zipinotti Dos Santos et al., 2023). Lipid droplets, composed of FAs, sterol esters, and triacylglycerols, encapsulated by a cholesterol and phospholipid monolayer, function as crucial subcellular organelles managing cellular lipid metabolism. Enzymes such as adipose triglyceride lipase (ATGL) regulate lipid catabolism on their surface (Yu et al., 2021; Farese and Walther, 2009; Olzmann and Carvalho, 2019; Tauchi-Sato et al., 2002).

## 3 Potential and obstacles in targeting lipid remodeling in breast cancer

**FIGURE 2:**
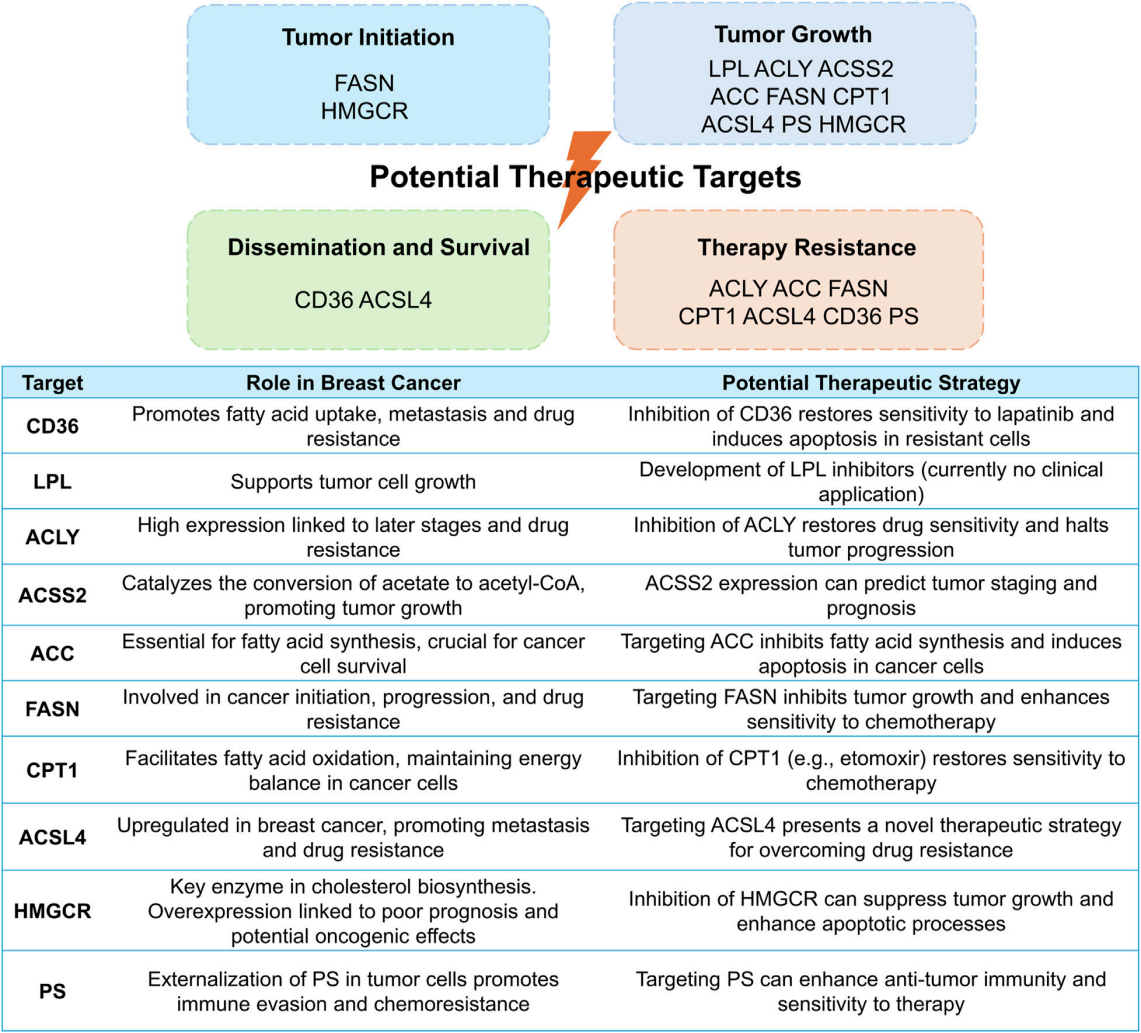
Potential therapeutic targets within lipid remodeling pathways in breast cancer: mechanisms, roles, and strategies. This figure elucidates diverse therapeutic targets in lipid remodeling, underlying tumorigenesis and progression (top), and underscores their significance in breast cancer, presenting potential strategies to leverage these pathways for therapeutic advantage (bottom).

The rapid proliferation of cancer cells heightens the necessity for local oxygen and nutrients, yet inadequately structured blood vessels fail to meet this demand. Consequently, the tumor microenvironment becomes acidic, hypoxic, and glucose-deprived. This triggers the activation and utilization of lipids as a primary energy source and key regulators within tumor cells, disrupting various signaling pathways and immune activities, while fostering tumor cell growth, proliferation, and migration (Zipinotti Dos Santos et al., 2023; Yu et al., 2021). Given that the landscape of lipid metabolism in breast cancer has been extensively covered in recent reviews (Xiao et al., 2024; Wang et al., 2024), our focus herein is primarily on the potential therapeutic targets within the lipid remodeling (Figure 2).

### 3.1 Fatty acid uptake

The overexpression of CD36 in cancer cells has reportedly been to enhance the transcription of genes associated with metastasis formation, facilitating the spread of cancer to lymph nodes in mice (Pascual et al., 2017). Feng and colleagues found that CD36 acted as a key player in resistance to lapatinib in human epidermal growth factor receptor 2 (HER2)-positive breast cancer cells, and inhibiting CD36 restored sensitivity to lapatinib and triggered apoptosis in those lapatinib-resistant cells (Feng et al., 2019). Moreover, elevated CD36 levels were found to be correlated with unfavorable prognoses (Pascual et al., 2017; Koundouros and Poulogiannis, 2020). Correspondingly, CD36 may provide a promising therapeutic target (Zhao J. et al., 2017). Besides, literature showed that LPL had the potential to accelerate the growth of tumor cells (Kuemmerle et al., 2011) However, no inhibitors targeting enzymes implicated in FA uptake have been introduced into clinical practice yet, restricting the translational potential of these findings.

### 3.2 Fatty acid synthesis

High expression of ACLY in breast cancer tissues is conversely proportional to disease stage and prognosis (Xiao et al., 2024; Chen et al., 2020). Furthermore, ACLY is implicated in the resistance to tamoxifen, palbociclib, and paclitaxel in breast cancer, whereas ACLY inhibition can re-establish sensitivity to these drugs and halts tumor progression (Chen et al., 2020; Ismail et al., 2022; Ismail et al., 2020; Velez et al., 2023). Besides cleavage of citrate by ACLY, the supply of cytosolic acetyl-CoA pool can be achieved via the conjugation of acetate with CoA under hypoxia and lipid-depleted conditions, catalyzed by acetyl-CoA synthetase 2 (ACSS2) (Schug et al., 2015). Hence, ACSS2 is essential for tumor growth under metabolic stress, and its expression can predict tumor staging and patient prognosis (Schug et al., 2015; Ling et al., 2022). As the rate-limiting enzyme of lipid biosynthesis, ACC definitely holds a pivotal position in breast cancer cell development (Brunet et al., 2008; Chajès et al., 2006), while silencing ACC hinders cell proliferation and triggers apoptosis in breast cancer cell lines (Chajès et al., 2006). Accordingly, ACC emerges as a potential target for suppressing FA biosynthesis in human cancers with pharmacological inhibitors exhibiting encouraging antitumor effects (Tong, 2005; Lally et al., 2019). As another crucial enzyme involved in DNL, FASN also contributes to breast cancer initiation, progression, and treatment resistance (Menendez and Lupu, 2017). Mechanistically, besides supplying energy to cancer cells, FASN exerts its pro-tumor effects through its positive feedback loop with HER2 (Menendez et al., 2021; Kumar-Sinha et al., 2003; Vazquez-Martin et al., 2008) as well as its interaction with nuclear factor-κB (NF-κB) (Liu et al., 2013; Wu et al., 2016), which affect downstream signaling pathways and cellular processes critical for cancer cell survival and resistance to treatment. Targeting FASN may therefore hold promise to inhibit tumor cell growth and enhance the vulnerability of breast cancer cells to traditional anticancer treatments (Menendez and Lupu, 2017).

### 3.3 Fatty acid oxidation

ACSs are categorized into five groups based on the length of the FA chain they act upon. Among them, long-chain ACSs (ACSLs) are accountable for the catalyzation of intracellular free long-chain FAs, which are then transported by transporter proteins such as CD36 (Rossi Sebastiano and Konstantinidou, 2019). Among the ACSL family, ACSL4 has been widely researched and linked to tumor progression. ACSL4 exhibits an abnormal upregulation in triple-negative breast cancer (TNBC) cells and is correlated with unfavorable prognosis and metastasis in TNBC patients (Qiu et al., 2024). Preclinical studies have revealed that ACSL4 can be activated in drug-resistant breast cancer cells (Li et al., 2022). In turn, this activation can impede the apoptotic pathway and enhance the mTOR pathway, thereby promoting cancer growth and conferring resistance to chemotherapy, endocrinotherapy, and radiotherapy (Li et al., 2022; Wu et al., 2013; Orlando et al., 2019). Consequently, targeting ACSL4 presents a novel therapeutic strategy for breast cancer, while transferring the basic scientific discoveries into clinical practice poses a significant hurdle. As the rate-limiting enzyme in FAO, CPT1 serves as a key factor in the initiation, progression, and migration of cancer cells (Wang et al., 2021). This enzyme not only fulfills the energy demands of cancer cells by facilitating FAO but also exerts its influence through various signaling pathways, cytokines, or microRNAs (Wang et al., 2021). Elevated levels of CPT1 expression have been detected in recurrent breast cancer cases, which are associated with unfavorable patient outcomes (Han et al., 2019). Moreover, serum CPT1 levels reliably signify the disease progression in breast cancer patients (Tan et al., 2021), underscoring its potential utility as a biomarker in breast cancer management. Accumulating evidence suggests that a key mechanism underlying CPT1’s role in fostering treatment resistance is its ability to sustain stemness in stem cells (Wang et al., 2018; Han et al., 2019; He et al., 2019; Shim et al., 2022; Chen et al., 2023). Preclinical studies have demonstrated that etomoxir (a CPT1 inhibitor) can re-sensitize multiple cancers, including breast cancer, to conventional chemotherapy and radiotherapy (Han et al., 2019; Shim et al., 2022; Chen et al., 2023; Tan et al., 2018; Pucer et al., 2013). The mechanism by which CPT2 mediates radiotherapy resistance in breast cancer cells is analogous to that of CPT1 (Han et al., 2019). Unfortunately, despite their potential, none of the FAO-related therapeutic targets have been successfully translated into clinical practice for breast cancer treatment.

### 3.4 Cholesterol synthesis

In cancer patients, there is an increase in endogenous cholesterol synthesis, coupled with elevated circulating cholesterol levels (Kopecka et al., 2020). The heightened cholesterol levels can augment downstream oncogenic pathway signaling, including Hedgehog- and mammalian target of rapamycin complex 1 (mTORC1)-dependent pathways (Ding et al., 2019). Furthermore, by exerting its influence on membrane fluidity, lipid raft function, and transcriptional processes, cholesterol can also regulate the activity of drug efflux transporters, thereby contributing to drug resistance (Kopecka et al., 2020; Ding et al., 2019; Celestino et al., 2015). The overexpression of MVA pathway-related molecules was observed in breast cancer patients with a worse prognosis (Kuzu et al., 2016; Brown et al., 2016; Clendening et al., 2010; Kim et al., 2019; Bjarnadottir et al., 2020). Additionally, HMGCR may possess oncogenic properties, and perturbations within the MVA pathway could facilitate cellular transformation (Clendening et al., 2010). Research has indicated that HMGCR and SQLE represent compelling targets for cancer treatment, albeit clinical studies on the latter are still lacking (Feltrin et al., 2020).

### 3.5 Phospholipids

Glycerophospholipids hold a critical position in the initiation and progression of cancer, and they mediate the resistance of cancer cells to chemotherapy via various mechanisms, encompassing modulating the composition of cellular membranes, influencing FA metabolism, acting as second messengers to activate DNA repair mechanisms, and upregulating drug efflux transporters (Kopecka et al., 2020). PS is a phospholipid confined to the inner leaflet of the cell membrane. In solid tumors, hypoxia and other pathophysiological stressors induce the externalization of PS, where it migrates to the outer leaflet (Gerber et al., 2018). The interaction between PS and its receptors critically regulates immuno-inflammatory responses, fostering an immune-evasive tumor microenvironment. Additionally, PS-receptor interaction mediates chemoresistance in cancer cells by the upregulation of drug efflux transporters and the activation of signaling cascades, e.g., phosphatidylinositol 3-kinase (PI3K)/Akt pathways (Zhou et al., 2020). These findings indicate the promising potential of PS-targeted therapy in combating tumor invasion and chemoresistance.

### 3.6 Obstacles in targeting lipid remodeling

As mentioned above, despite the existence of numerous promising targets, their successful clinical translation remains elusive. Presently, the therapeutic strategies targeting lipid metabolism in breast cancer face several obstacles that require attention. Firstly, the inhibition of lipid metabolic pathways may raise safety concerns due to their potential to induce detrimental effects on normal cells, thereby limiting the development of safe and specific inhibitors (Ligorio et al., 2021). Secondly, the translation of preclinical findings into a clinical setting poses significant challenges, as experiment conditions utilized in preclinical studies may employ supra-physiological concentrations of lipid-modulating drugs (Goodwin et al., 2022). Thirdly, the metabolic adaptability of cancer cells may undermine the effectiveness of inhibiting a single enzyme or pathway alone in sustaining long-term tumor suppression (Ligorio et al., 2021; Fendt et al., 2020). Notwithstanding the challenges, some therapeutic strategies targeting lipid metabolism have still progressed into clinical trials, which will be exhaustively presented in the following section.

## 4 Clinical trials concerning regulation of lipid metabolism in patients with breast cancer

### 4.1 Anticancer activity

Despite strong evidence supporting FASN as a promising target for breast cancer therapy, only a few compounds inhibiting FASN have entered clinical studies to date. TVB-2640 is a small molecule that stands as the first highly selective human FASN inhibitor to progress into clinical trials (Falchook et al., 2021). A phase I clinical trial have confirmed the biological activities and safety of the TVB-2640 in solid tumors, including breast cancer (Falchook et al., 2021). Accordingly, an ongoing Phase II clinical study (NCT03179904) will further investigate the efficacy and safety profile of TVB-2640 administered in conjunction with trastuzumab plus paclitaxel or endocrine therapy in the management of advanced HER2-positive breast cancer (Table 1). Besides the FASN inhibitor TVB-2640, several other clinical medications or plant-derived polyphenols have been demonstrated to block FASN, among other targets, and enhance the effectiveness of numerous traditional therapies. Examples include omeprazole (Sardesai et al., 2021), conjugated linoleic acid (CLA) (McGowan et al., 2013), and epigallocatechin-3-gallate (EGCG) (Brusselmans et al., 2003), which have undergone corresponding clinical trials. Notably, research results for the former two have been published (Table 2). Consequently, the combination of omeprazole and neoadjuvant chemotherapy has yielded a promising pathological complete response (pCR) rate (Sardesai et al., 2021), and preoperative administration of CLA for ten to 28 days resulted in significant reductions in Spot 14 and Ki67 levels (McGowan et al., 2013).

**TABLE 1:** Completed (unpublished) and ongoing trials of lipid pathway-targeted therapy in breast cancer (BC).

Target	Inhibitor	Trial number	Phase	Enrollment (actual)	Setting	Regimen	Primary endpoint	Status
FASN	TVB-2640	NCT03179904	II	19	Taxane and trastuzumab-resistant, HER2+ ABC	TVB-2640 + trastuzumab + paclitaxel/endocrine therapy	ORR	Ongoing
	EGCG	NCT05680662	I	200	Any type of BC	Arm A: quercetin + zinc + EGCG + metformin + chemotherapy	iDFS	Ongoing
						Arm B: chemotherapy		
HMGCR	Atorvastatin	NCT03872388	II	6	Stage IIb-III TNBC who did not achieve a pCR after receiving NACT	Group A: atorvastatin	The proportion of CTC-negative	Terminated
						Group B		
						Arm a: capecitabine		
						Arm b: none		
		NCT01980823	I	23	EBC, WOO	Atorvastatin + metformin	Ki67	Completed
		NCT04601116	III	3,360	ER + EBC	Arm A: (neo)adjuvant therapy + atorvastatin	iDFS	Ongoing
						Arm B: (neo)adjuvant therapy + placebo		
		NCT02416427	II	78	TAZ-expressing EBC, WOO	Atorvastatin	Ki67	Unknown
		NCT05103644	II/III	60	HER2- BC	Arm A: atorvastatin	Ki67, TAZ expression, and cardiac markers	Ongoing
						Arm B: placebo		
		NCT02958852	II	126	ER+/HER2- ABC	Arm A: letrozole + atorvastatin	CBR	Unknown
						Arm B: letrozole^a^		
		NCT05507398	Ⅳ	100	Non-metastatic BC	Arm A: atorvastatin + NACT	ORR and pCR	Unknown
						Arm B: metformin + NACT		
						Arm C: placebo + NACT		
		NCT03358017	II	54	TNBC	Arm A: NACT + zoledronate + atorvastatin	pCR and YAP/TAZ expression	Completed
						Arm B: NACT		
	Simvastatin	NCT05550415	II	26	Advanced TNBC	Arm A: chemotherapy + simvastatin	Vimentin epression	Ongoing
						Arm B: chemotherapy + placebo		
		NCT05464810	I	40	Postmenopausal non-metastatic HR+/HER2- BC, WOO	Arm A: letrozole + simvastatin	Ki67	Ongoing
						Arm B: letrozole		
		NCT03324425	II	5	HER2+ ABC	Simvastatin + dual anti-HER2 Therapy	ORR	Ongoing
		NCT03192293	II	28	Postmenopausal ER + ABC	Simvastatin + metformin + fulvestrant	CBR	Unknown
ACC	Metformin^b^	NCT01589367	II	208	Non-diabetic, postmenopausal ER + BC	Arm A: neoadjuvant letrozole + metformin	CRR	Completed
						Arm B: neoadjuvant letrozole + placebo		
		NCT01566799	II	60	LABC	Metformin + NACT	pCR	Unknown
		NCT04387630	II/III	120	Non-diabetic BC	Arm A: metformin + NACT	CRR	Unknown
						Arm B: placebo + NACT		
		NCT03238495	II	100	HER2+ EBC	Arm A: metformin + NACT + FMD	pCR	Unknown
						Arm B: NACT + FMD		
		NCT04248998	II	30	TNBC	Arm A: metformin + NACT	pCR	Ongoing
						Arm B: NACT		
		NCT05023967	II	120	HR + EBC, WOO	Arm A: metformin + FMD	Safety and Ki67	Ongoing
						Arm B: usual dietary		
		NCT01477060	II	32	HR+/HER2- ABC with progressive disease after first-line therapy	Arm A: hormonal therapy + metformin	PFS	Terminated
						Arm B: hormonal therapy + lapatinib		
						Arm C: hormonal therapy + Metformin + lapatinib		
		NCT04143282	II	250	ABC	Arm A: metformin + chemotherapy	ORR, OS, and PFS	Completed
						Arm B: chemotherapy		
		NCT02506777	II	96	LABC	Arm A: NACT + metformin	ORR and pCR	Unknown
						Arm B: NACT + melatonin		
						Arm C: NACT		
		NCT02506790	II	96	LABC	Arm A: neoadjuvant toremifene + metformin	ORR and pCR	Unknown
						Arm B: neoadjuvant toremifene + melatonin		
						Arm C: neoadjuvant toremifene		
PS	Bavituximab	NCT00669565	II	46	LABC or ABC	Bavituximab + paclitaxel + carboplatin	ORR	Completed
		NCT00669591	II	46	ABC	Bavituximab + docetaxel	ORR	Completed

FASN, fatty acid synthase; HER2, human epidermal growth factor receptor 2; ABC, advanced breast cancer; ORR, overall response rate; EGCG, epigallocatechin-3-gallate; BC, breast cancer; iDFS, invasive disease-free survival; HMGCR, 3-hydroxy-3-methylglutaryl-coenzyme A reductase; TNBC, triple-negative breast cancer; pCR, pathological complete response; NACT, neoadjuvant chemotherapy; CTC, circulating tumor cell; EBC, early breast cancer; WOO, window-of-opportunity; ER, estrogen receptor; TAZ, transcriptional co-activator with PDZ-binding motif; CBR, clinical benefit rate; YAP, Yes-associated protein; HR, hormone receptor; ACC, acetyl-CoA carboxylase; CRR, clinical response rate; LABC, locally advanced breast cancer; FMD, fasting-mimicking diet; OS, overall survival; PFS, progression-free survival; PS, phosphatidylserine.

^a^ Fulvestrant will be used as second line endocrine treatment upon progression on first line with letrozole ± atorvastatin.

^b^ Some studies related to metformin have already been mentioned in the section on statins.

**TABLE 2:** Published trials concerning lipid pathway-targeted therapy in breast cancer (BC).

Target	Inhibitor	Phase	No.	Setting	Regimen	Primary endpoint	Findings	References
FASN	Omeprazole	II	42	Early TNBC	NACT + omeprazole	pCR	Yielded a promising pCR rate	Sardesai et al. (2021)
	CLA	I	24	EBC, WOO	CLA	Expression of biomarkers	Significant decrements in Spot 14 and Ki67	McGowan et al. (2013)
HMGCR	Fluvastatin	NA	40	Stage 0/I BC, WOO	Fluvastatin	Ki67	Reduced tumor proliferation and increased apoptotic activity in high-grade tumors	Garwood et al. (2010)
	Atorvastatin	II	50	Postmenopausal BC, WOO	Atorvastatin	Ki67	Indicated MAPK pathway inhibition, and pro-apoptotic and anti-proliferative effects	Feldt et al. (2015), Bjarnadottir et al. (2015) Feldt et al. (2020))
	Simvastatin	II	24	Stage I/II BC, WOO	Simvastatin	Ki67	Ki-67 remained unchanged, whereas significant increases were detected in apoptotic markers	Kamal et al. (2024)
		II	66	LABC	Arm A: NACT + placebo	Clinical response	Improved ORR and pathological response, especially in patients with HER2 overexpression	Yulian et al. (2021), Yulian et al. (2023)
					Arm B: NACT + simvastatin			
	Pitavastatin	II/III	70	EBC	Arm A: NACT + placebo	CRR and Ki67	Higher reductions in tumor size	Dewidar et al. (2022)
					Arm B: NACT + pitavastatin			
ACC	Metformin	II	60	Pre-treated postmenopausal HR + ABC	Arm A: AI + metformin	PFS	No improved efficacy	Zhao et al. (2017b)
					Arm B: AI + placebo			
		II	122	Non-diabetic HER2- ABC	Arm A: metformin + chemotherapy	PFS	The addition of metformin did not improve PFS, whereas alleviated chemotherapy toxicity. Patients with insulin resistance exhibited significantly shortened PFS.	MYME (Nanni et al., 2019)
					Arm B: chemotherapy			
		NA	47	Non-diabetic EBC, WOO	Arm A: metformin	Ki67	Ki67 fell significantly	Hadad et al. (2011), Hadad et al. (2015)
					Arm B: none			
		II	80	Non-diabetic LABC	Arm A: metformin + NACT	CBR	Improveed non-significantly the clinical and pathological tumor response	Barakat et al. (2022)
					Arm B: NACT			
		II	35	Overweight EBC, WOO	Metformin	Ki67	There was no reduction in Ki67	Kalinsky et al. (2014)
		NA	39	Non-diabetic EBC, WOO	Metformin	Ki67	Insulin-dependent effects of metformin as its antitumor mechanism	Dowling et al. (2015)
		II	22	Postmenopausal, overweight HR+/HER2- ABC	Everolimus + exemestane + metformin	PFS	Safe and had moderate clinical benefit	Yam et al. (2019)
		II	92	EBC with metabolic abnormality	Arm A: Metformin + NACT	pCR	Did not increase pCR rate	NeoMET (Huang et al., 2023)
					Arm B: NACT			
		NA	107	Non-diabetic ABC	Arm A: Metformin + chemotherapy	PFS and RR	No significant survival benefit	Essa et al. (2022)
					Arm B: chemotherapy			
		II	40	ABC	Arm A: Metformin + standard chemotherapy	PFS	No significant effect on RR, PFS, or OS	Pimentel et al. (2019)
					Arm B: Placebo + standard chemotherapy			
		II	70	EBC	Arm A: NACT + Metformin	Apoptosis biomarker and safety	Improved clinical and pathological responses, alleviated chemotherapy toxicity	METNEO (Serageldin et al., 2023; Serageldin et al., 2024)
					Arm B: NACT			
		III	3,649	Non-diabetic T1c-3N0-3M0 BC	Arm A: metformin	iDFS	Did not significantly improve iDFS; did not reduce the risk of new cancer development	MA.32 (Pimentel et al., 2021; Goodwin et al., 2015; Goodwin et al., 2021a; Goodwin et al., 2021b; Goodwin et al., 2022; Goodwin et al., 2023)
					Arm B: placebo			
		II	84	Non-metastatic HER2+ BC	Arm A: metformin + NACT	pCR	Did not significantly improve pCR rate	METTEN (Martin-Castillo et al., 2018; Cuyàs et al., 2019a; Cuyàs et al., 2019b; Lopez-Bonet et al., 2019)
					Arm B: NACT			
		II	59	Stage II/III non-diabetic BC	Arm A: metformin + NACT	Pathological response	Did not significantly improve pCR rate	El-Khayat et al. (2021)
					Arm B: NACT			
		II	200	Non-diabetic EBC, WOO	Arm A: metformin	Ki67	Did not significantly affect Ki67 overall, but showed significantly different effects according to insulin resistance, particularly in luminal B tumors	Bonanni et al. (2012), Cazzaniga et al. (2013), DeCensi et al. (2015)
					Arm B: placebo			
		II	39	Non-diabetic BC, WOO	Metformin	Ki67	Ki67 staining in invasive tumor tissue decreased and TUNEL staining increased	Niraula et al. (2012)
		II	47	Non-diabetic HER2+ EBC/LABC	Metformin + NACT	pCR	Did not appear to improve activity over conventional sequential regimens	Rocca et al. (2021)
PS	Bavituximab	I	14	HER2- ABC	Bavituximab + paclitaxel	Safety	Well tolerated	Chalasani et al. (2015)

FASN, fatty acid synthase; TNBC, triple-negative breast cancer; NACT, neoadjuvant chemotherapy; pCR, pathological complete response; CLA, conjugated linoleic acid; EBC, early breast cancer; WOO, window-of-opportunity; HMGCR, 3-hydroxy-3-methylglutaryl-coenzyme A reductase; BC, breast cancer; LABC, locally advanced breast cancer; ORR, objective response rate; HER2, human epidermal growth factor receptor 2; CRR, clinical response rate; ACC, acetyl-CoA carboxylase; HR, hormone receptor; PFS, progression-free survival; ABC, advanced breast cancer; CBR, clinical benefit rate; RR, response rate; OS, overall survival; iDFS, invasive disease-free survival; TUNEL, terminal deoxynucleotidyl transferase-mediated dUTP, nick end labeling.

Statins, functioning via the inhibition HMGCR, are commonly prescribed for patients with cardiovascular disease and high cholesterol levels to lower their cholesterol (Clendening and Penn, 2012). In recent years, growing trials have focused on their promising anticancer benefits (Kamal et al., 2024; Yulian et al., 2021; Dewidar et al., 2022; Yulian et al., 2023; Garwood et al., 2010; Feldt et al., 2015; Bjarnadottir et al., 2015; Nielsen et al., 2012). Currently, clinical trials for drugs such as fluvastatin, atorvastatin, simvastatin, and pitavastatin have been initiated worldwide, primarily for use before surgery in early breast cancer. The potential of these drugs, either as monotherapy or in addition to traditional standard therapy, is being evaluated through clinical or pathological responses (Tables 1, 2). Among them, results from three window-of-opportunity studies have been published, generally finding that short-term monotherapy with statins could suppress tumor growth and enhance apoptotic processes (Kamal et al., 2024; Garwood et al., 2010; Feldt et al., 2015; Bjarnadottir et al., 2015). Additionally, two published studies in the neoadjuvant setting suggested that statins combined with standard neoadjuvant chemotherapy could, to some extent, enhance clinical and pathological responses compared to placebo, although the statistical differences were not always significant (Yulian et al., 2021; Dewidar et al., 2022; Yulian et al., 2023). Ongoing trials will delve deeper into the therapeutic potential of statins as monotherapy, alongside chemotherapy or with endocrine therapy for early- and advanced-stage breast cancer. For instance, the ongoing Phase III MASTER trial (NCT04601116) aims to determine whether long-term statin therapy can enhance the prognosis of women with early breast cancer.

Metformin, a frequently prescribed biguanide, stands as a cornerstone medication in managing hyperglycemia and type 2 diabetes (Pernicova and Korbonits, 2014). Increasing evidence suggests a potential effectiveness of this compound as an anticancer agent (Pernicova and Korbonits, 2014; Thompson, 2014). A fundamental way in which metformin exerts its influence encompasses the stimulation of AMP-activated protein kinase (AMPK), a vital metabolic modulator essential for maintaining energy balance and regulating cellular growth (Ben Sahra et al., 2010). Of note, AMPK has an impact on inhibiting ACC activity (Fullerton et al., 2013; Svensson et al., 2016). Accordingly, the antitumor activity of metformin could be at least partially facilitated by hindering FA biosynthesis (Ligorio et al., 2021). Herein, we also provide an overview of the clinical studies regarding the application of metformin in breast cancer (Table 1 and 2). Unfortunately, the research findings remain controversial. Some studies have found that preoperative monotherapy with metformin for 2-4 weeks could significantly reduce Ki67 levels and oncogenic signaling (Hadad et al., 2011; Dowling et al., 2015; Niraula et al., 2012; DeCensi et al., 2014). Furthermore, a study conducted by Serageldin et al. demonstrated that metformin could provide an additional benefit in terms of clinical and pathological responses compared to neoadjuvant chemotherapy alone (Serageldin et al., 2024). However, most research, including a phase III trial (MA.32) involving 3,649 patients (Goodwin et al., 2022), has indicated that metformin offered no advantage in antitumor effect, whether in early-stage (El-Khayat et al., 2021; Goodwin et al., 2022; Goodwin et al., 2023; Barakat et al., 2022; Kalinsky et al., 2014; Huang et al., 2023; Martin-Castillo et al., 2018; Cuyàs et al., 2019a; Cuyàs et al., 2019b; Lopez-Bonet et al., 2019; Bonanni et al., 2012; Cazzaniga et al., 2013; DeCensi et al., 2015; Rocca et al., 2021) or late-stage (Zhao Y. et al., 2017; Essa et al., 2022; Nanni et al., 2019; Pimentel et al., 2019; Yam et al., 2019) breast cancer, and regardless of whether the treatment was administered as monotherapy or in addition to conventional regimens. Notably, preclinical studies on ACC inhibition have exhibited contradictory outcomes regarding its anticancer effectiveness (Ligorio et al., 2021). Furthermore, Liu et al. discovered that the conjunction of metformin and simvastatin exhibits synergistic inhibition of various cancer cells, suggesting its potential in antitumor therapy when used in conjunction (Liu et al., 2023).

Bavituximab represents an unconjugated chimeric immunoglobulin G1 (IgG1) monoclonal antibody specifically binds to PS (Gerber et al., 2018). The unique external exposure of PS on tumor cells makes bavituximab an effective agent for targeting tumor specifically (Jennewein et al., 2008). Preclinical studies have shown that chemotherapy and radiation therapy could increase the exposure of PS, further enhancing bavituximab binding and immune activation (Huang et al., 2005; He et al., 2007). Therefore, bavituximab may serve as an ideal partner for chemotherapy and radiation therapy. A phase I clinical trial has thus far confirmed the safety of bavituximab administration in breast cancer (Chalasani et al., 2015). Additionally, two ongoing phase Ⅱ trials (NCT00669565 and NCT00669591) have completed their investigation into the potential value of combining bavituximab with chemotherapy in advanced breast cancer, although the research data have not yet been disclosed (Table 1). However, a randomized phase III trial comparing docetaxel plus bavituximab to docetaxel alone in patients with previously treated advanced non-squamous non-small-cell lung cancer failed to demonstrate survival benefit for the combination therapy (Gerber et al., 2018).

### 4.2 Complication reduction and cancer prevention potential

Despite contradictory research findings regarding its direct anticancer effects in breast cancer, lipid pathway-targeted therapy has been widely accepted for its crucial role in managing complications associated with treatments such as chemotherapy. For instance, metformin has been shown to alleviate chemotherapy-induced peripheral neuropathy neutropenia, cardiac toxicity, oral mucositis, and fatigue in non-diabetic breast cancer patients (Serageldin et al., 2023; Nanni et al., 2019; Bakry et al., 2023). Based on the preclinical evidence, statins appear to have the potential to mitigate chemotherapy-induced cardiotoxicity (Padegimas et al., 2020; Pecoraro et al., 2023; Bjørnstad et al., 2022). However, results from published clinical studies indicate that statins did not affect declines in left ventricular ejection fraction among certain breast cancer patients (Hundley et al., 2022; Makhlin et al., 2024; Thavendiranathan et al., 2023). An ongoing study is currently assessing the use of atorvastatin for prophylactic cardioprotection in patients undergoing anti-HER2 targeted therapy (NCT05559164). Lipid pathway-targeted therapy is also capable of protecting patients from the side effects of radiation therapy. In a phase Ⅱ double-blind, placebo-controlled randomized trial, the prophylactic application of EGCG solution significantly decreased both the occurrence and magnitude of radiation-induced dermatitis among breast cancer patients receiving adjuvant radiotherapy (Zhao et al., 2022). Statins are also believed to alleviate radiation-related complications (Ricco and Kron, 2023), however, clinical studies related to statin protection during radiotherapy in breast cancer patients have been terminated (NCT04385433, NCT00902668).

Furthermore, there are currently numerous completed as well as ongoing clinical trials exploring the potential of lipid pathway-targeted therapy in breast cancer prevention. The omega-3 preparation Lovaza was reported to reduce breast cancer risk in obese women (Manni et al., 2017). Although many observational analyses have found that metformin therapy did not practically influence cancer incidence (Dankner et al., 2019; Dickerman et al., 2023), there are also studies that supported a potential beneficial impact of metformin on reducing cancer risk (Evans et al., 2005; Muszyńska-Ogłaza et al., 2017; Tapia et al., 2021; Tseng, 2014). A clinical trial that is still ongoing will specifically explore the potential of metformin in preventing the development of invasive breast cancer among patients with a history of breast carcinoma *in situ* or atypical hyperplasia (NCT01905046). Similarly, there is no definitive conclusion pertaining to the association between statin administration and breast cancer risk (Higgins et al., 2012; Vinayak et al., 2013). Of note, several studies have indicated that lipophilic statins (i.e., simvastatin, fluvastatin, and lovastatin) may offer superior breast cancer prevention benefits compared to hydrophilic statins (i.e., atorvastatin and pravastatin) (Ahern et al., 2011; Cauley et al., 2006).

### 4.3 Selective lipid metabolism therapy benefit hypothesis

Based on the above, the clinical evidence projecting lipid pathway-targeted therapy as an anticancer or cancer chemopreventive strategy is variable. Nonetheless, it is recognized that certain breast cancer patients can indeed benefit from lipid pathway-targeted therapy (Serageldin et al., 2024; El-Khayat et al., 2021; Kamal et al., 2024; Yulian et al., 2021; Dewidar et al., 2022; Yulian et al., 2023; Serageldin et al., 2023). Consequently, we advance the Selective Lipid Metabolism Therapy Benefit Hypothesis, suggesting that therapeutic strategies targeting lipid metabolism may selectively benefit a subset of patients. This hypothesis neither unconditionally advocates for the absolute benefits of targeting lipid metabolism nor conclusively denies its significance based solely on negative trial results. Instead, it offers a more objective and dialectical understanding of the clinical value of lipid pathway-targeted therapies. This hypothesis highlights the need for a more precise identification of the patient subpopulation that may potentially benefit from such treatment, which is of paramount importance. Currently, some clinical trials have identified potential biomarkers capable of predicting the treatment response to lipid metabolism-targeting therapies, which will be comprehensively summarized in the next section.

### 4.4 Selective biomarkers

Currently, a number of clinical trials have identified a panel of promising biomarkers (Feldt et al., 2015; Cuyàs et al., 2019a; Hadad et al., 2015; Pimentel et al., 2021; Goodwin et al., 2015; Goodwin et al., 2021a; Feldt et al., 2020; Ahern et al., 2020; Goodwin et al., 2021b). Based on an exploratory analysis of a window-of-opportunity breast cancer trial, Feldt and colleagues suggested that the expression levels of cyclin D1, p27, and low-density lipoprotein receptor in tumor tissues might influence the anti-proliferative efficacy of atorvastatin (Feldt et al., 2015; Feldt et al., 2020). Furthermore, evidence exists indicating that HER2 overexpression and genetic variations in ABCB1, CYP3A4, SLCO1B1, and CYP3A5 serve as biomarkers for predicting the response of breast tumors to simvastatin (Yulian et al., 2021; Ahern et al., 2020). Regarding Metformin, studies have found it to be more effective in patients with a BMI ≥25 (El-Khayat et al., 2021), although this finding remains controversial (Kalinsky et al., 2014). The Phase Ⅱ METTEN trial suggested that the C allele of ataxia telangiectasia mutated (ATM) rs11212617 might function as a predictive biomarker to guide the personalized use of metformin in patients with breast cancer (Cuyàs et al., 2019a); however, subsequent findings from the Phase Ⅲ MA.32 trial refuted this notion (Goodwin et al., 2021b). This controversy may stem from differences in the patient populations included in the two studies, as some research indicates that the anticancer effects of metformin in breast cancer exhibit subtype specificity (Yulian et al., 2021; Bonanni et al., 2012). Specifically, the METTEN trial exclusively enrolled HER2-positive breast cancer patients, whereas the MA.32 trial included patients regardless of their subtype. Additionally, the METTEN trial proposed that serum homocysteine levels might serve as an indicator connecting the antifolate-mimicking effects of metformin with tumor response (Cuyàs et al., 2019b). An observable trend towards differing metformin efficacy, based on insulin resistance status, is also noted (Bonanni et al., 2012; Cazzaniga et al., 2013). Further exploration and validation of stable biomarkers for lipid pathway-targeted therapy in large-scale clinical trials are warranted.

## 5 Conclusion and perspectives

The reprogramming of lipid metabolism has a vital role in breast cancer progression, drug resistance, and clinical outcome. We herein elaborate on the potential of regulating lipid metabolism in the clinical application for breast cancer, focusing on key enzymes and clinical trials. However, contrary to expectations, many trials regarding lipid pathway-targeted therapy have yielded negative results, possibly due to patient heterogeneity and the intricate crosstalk between lipid metabolism and cancer biology. Therefore, we propose the Selective Lipid Metabolism Therapy Benefit Hypothesis, advocating for the development of predictive biomarkers to guide personalized treatment decisions. Future research should focus on identifying and validating these biomarkers, as well as exploring novel therapeutic strategies to target lipid metabolism in breast cancer patients. By optimizing lipid pathway-targeted therapy, we can potentially improve patient outcomes, reduce complications, and prevent cancer recurrence.

## Data Availability

The original contributions presented in the study are included in the article/supplementary material, further inquiries can be directed to the corresponding authors.
